# An Unusual Case of Gastrointestinal Bleeding from Isolated Gallbladder Varices in a Patient with Pancreatic Cancer Complicated by Portal Biliopathy

**DOI:** 10.1155/2016/8602378

**Published:** 2016-10-05

**Authors:** Mahir Gachabayov, Kubach Kubachev, Elbrus Abdullaev, Nonna Zarkua, Abakar Abdullaev, Artur Fokin

**Affiliations:** ^1^Department of Abdominal Surgery, Vladimir City Clinical Hospital of Emergency Medicine, Vladimir, Russia; ^2^Department of Surgery Named after N. D. Monastyrskiy, North-Western State Medical Academy Named after I. I. Mechnikov, Saint Petersburg, Russia

## Abstract

Portal biliopathy is the complex of abnormalities of extrahepatic and intrahepatic bile ducts, cystic duct, and gallbladder, arising as a result of extrahepatic portal vein obstruction and noncirrhotic portal fibrosis, which can be caused by coagulopathies, tumors, inflammation, postoperative complications, dehydration, and neonatal umbilical vein catheterization. We report a case of a 55-year-old male patient with the history of pancreatic cancer and cholecystoenteric anastomosis presenting with gastrointestinal bleeding from gallbladder varices via the anastomosis.

## 1. Introduction

Portal biliopathy (PB) is the complex of abnormalities of extrahepatic and intrahepatic bile ducts, cystic duct, and gallbladder, arising as a result of extrahepatic portal vein obstruction (EHPVO) and noncirrhotic portal fibrosis, which can be caused by coagulopathies, tumors, inflammation, postoperative complications, dehydration, and neonatal umbilical vein catheterization [1–4]. Although jaundice and common bile duct (CBD) compression associated with portal hypertension were described by Fraser et al. in 1944, the term “portal biliopathy” was not used until the early 1990s [2, 5, 6]. The incidence of PB is unknown due to its rarity. However, ectopic varices were estimated to be encountered in 12–30% in the field of portal hypertension [7]. They are usually located in the gallbladder and bile ducts where they can coexist and are usually asymptomatic [8, 9]. Few reports have been published on this topic. We herein report an extremely rare case of gastrointestinal bleeding as a result of bleeding from gallbladder varices via cholecystoenteric anastomosis caused by pancreatic carcinoma.

## 2. Case Presentation

**Figure 1:**
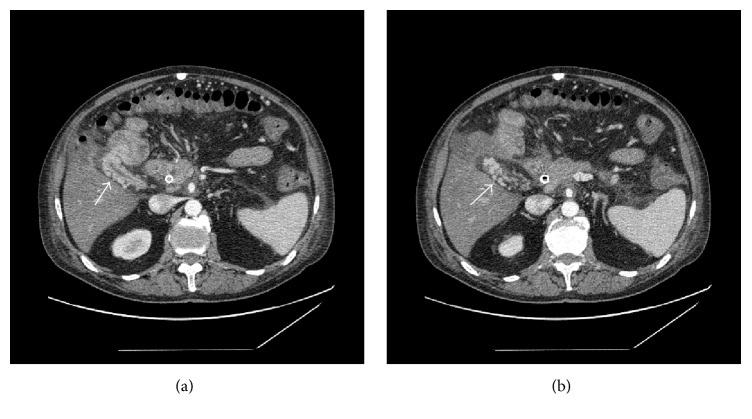
Abdominal contrast-enhanced CT, axial plane, showing gallbladder varices (diffuse variceal dilation of veins in the gallbladder wall).

**Figure 2:**
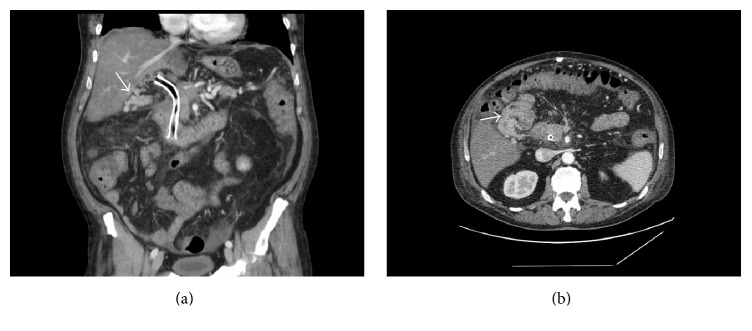
Abdominal contrast-enhanced CT. (a) Oblique plane, showing gallbladder varices, and (b) axial plane, showing gallbladder varices and cholecystoenteric anastomosis.

**Figure 3:**
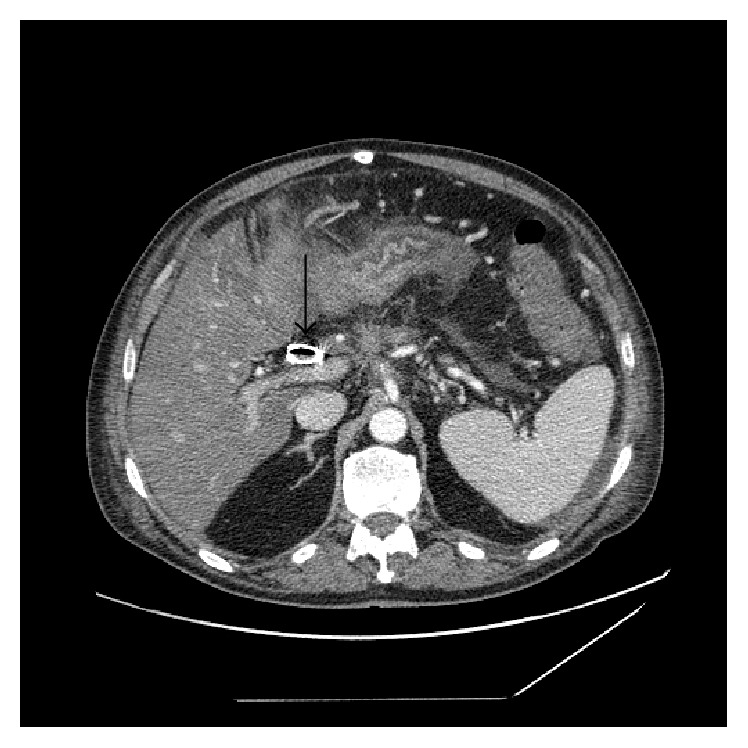
Abdominal contrast-enhanced CT, axial plane. Stented common bile duct is intimately close to the portal vein causing its minor compression.

A 55-year-old male patient was admitted to ICU with a 9-hour history of melena, fatigue, and nausea. His past medical history was significant for unresectable Stage 3 pancreatic adenocarcinoma (T4N1M0) which was proven during explorative laparotomy 4 months prior to admission. At that laparotomy preventive cholecystoenteric anastomosis was formed. Nevertheless, the patient was reported to develop jaundice for which choledochal stent was placed a month later. He also had well-controlled hypertension and peptic ulcer disease. On admission, the patient was pale and hypotonic, with HR 96 bpm, BP 105/60 mmHg, Hb 8.0 g/dL, serum amylase 94 U/L, total bilirubin 0.8 mg/dL, and direct bilirubin 0.2 mg/dL. Fluid replacement therapy, PPIs, and hemostatic agents were started. Esophagogastroduodenoscopy was negative for both the source and signs of bleeding while colonoscopy after preparation with laxatives was positive for signs of gastrointestinal bleeding and negative for source. Contrast-enhanced CT was performed thereafter which revealed an extremely rare picture of isolated gallbladder varices bleeding to the gastrointestinal tract via cholecystoenteric anastomosis (Figures 1 and 2). The patient underwent laparotomy with disconnection of cholecystoenteric anastomosis. The gallbladder seemed to be difficult to remove due to the carcinogenic infiltration of its neck, so the gallbladder wall was repaired. The etiology of portal biliopathy with isolated gallbladder varices was deemed to be combined: due to the infiltration (either inflammatory or carcinogenic) of small branches of portal vein and the minor compression of portal vein by the stented common bile duct (Figure 3). On the 3rd postoperative day the patient developed ileus which was resolved conservatively. On the 8th postoperative day surgical site infection without major septic consequences developed which was also resolved conservatively. The patient was discharged on 14th postoperative day. On the follow-up after 1 month the patient was relatively well without any episodes of recurrent bleeding and jaundice but with the progression of tumor intoxication.

## 3. Discussion

Ectopic varices are dilated mesoportal veins and/or portosystemic collateral that can occur along the entire gastrointestinal tract outside the common pathologic variceal sites. They represent 2–5% of gastrointestinal variceal bleeding; however, they are associated with a 4-fold increased risk of bleeding when compared with esophageal varices and can have a mortality rate as high as 40% [10]. Ectopic varices are commonly associated with portal hypertension in the context of liver cirrhosis or portal vein thrombosis [11]. Several cases of isolated varices of different sites including one-sided portal hypertension, isolated small bowel, large bowel, or duodenal varices have been reported [12–15].

The drainage vein of the gallbladder, the cystic vein, in some cases can be prominent, accompanying the cystic duct and ending in the right branch of the portal vein; in some cases they can drain blood from the gallbladder directly to the liver parenchyma [16]. The drainage veins of biliary ducts form epicholedochal and paracholedochal plexuses (Petren's plexus) running along the CBD [17, 18]. Portal vein thrombosis or compression can lead to the development of new collateral, thus, forming portal cavernoma and varicosity of cystic veins and epi- and paracholedochal plexuses [19]. Pancreatic cancer has been reported and is well-recognized to be one of the causes of PB [20].

Clinical presentation of PB is diverse varying from asymptomatic to obstructive jaundice and cholangitis. Symptomatic PB is rare, with estimates ranging between 5% and 38% of patients with portal vein thrombosis [17, 19, 21, 22]. In 2–4% of cases, patients with long-term obstruction or inadequate endoscopic or surgical management may develop secondary biliary cirrhosis [1]. Among complications of PB, obstructive syndrome with its consequences is more specific for CBD varices, while gallbladder varices can be complicated by bleeding and even rupture [23]. Bleeding from the gallbladder varices can occur either to the peritoneal cavity leading to a life-threatening hemoperitoneum or to the cavity of gallbladder itself leading to hemobilia and gastrointestinal bleeding [8, 9, 20, 24, 25]. In our case gallbladder varices were bleeding to the cavity of the gallbladder itself; however, the route of blood transmission emerged to be cholecystoenteric anastomosis instead of major duodenal papilla and hemobilia. To the best of our knowledge, our case is the second case of bleeding gallbladder varices via the anastomosis leading to major gastrointestinal bleeding [26].

Some authors support duplex scan and color flow Doppler sonography to be the gold standard for PB showing anechoic, serpentine areas in the wall or around the gallbladder with venous flow on Doppler imaging [27, 28]. Contrast-enhanced ultrasound has been shown to be more sensitive and accurate to distinguish between vascular structures and solid masses [3]. Although endoscopic ultrasonography has been shown to add to the differentiation between CBD varices, stones, and tumors in cases when other radiologic modalities failed, it is not routinely recommended in the workup of the patient with PB [29]. Contrast-enhanced CT is also helpful in the diagnosis of gallbladder varices being accurate in the imaging of vascular structures [30, 31]. MRCP features of PB have been shown to be biliary stenosis, wavy appearance of the bile ducts, angulation of the CBD, and upstream dilatation of the bile ducts [32].

Asymptomatic cases of PB do not require any treatment. Portosystemic shunt surgery seems to be the procedure of choice in patients with radiologic findings of PB who are otherwise asymptomatic and are being operated on for other complications of portal hypertension, such as variceal bleeding or symptomatic hypersplenism [33]. In emergency cases, especially in patients with life-threatening intra-abdominal or gastrointestinal bleeding, the vector shifts toward simplicity and minimal invasiveness [26].

To conclude, portal biliopathy is a rare but potentially life-threatening clinical entity which should be considered in differential diagnosis in patients with the history of pancreatic cancer. High index of clinical suspicion, multitechnique imaging approach, and prompt and adequate treatment are essential for the successful management of patients with portal biliopathy.

## References

[1] Chandra R., Kapoor D., Tharakan A., Chaudhary A., Sarin S. K. (2001). Portal biliopathy. *Journal of Gastroenterology and Hepatology*.

[2] Sarin S. K., Bhatia V., Makwane U. (1992). Portal biliopathy in extra hepatic portal vein obstruction. *Indian Journal of Gastroenterology*.

[3] Nunoi H., Hirooka M., Ochi H. (2013). Portal biliopathy diagnosed using color Doppler and contrast-enhanced ultrasound. *Internal Medicine*.

[4] Pargewar S. S., Desai S. N., Rajesh S., Singh V. P., Arora A., Mukund A. (2016). Imaging and radiological interventions in extra-hepatic portal vein obstruction. *World Journal of Radiology*.

[5] Fraser J., Broun A. K. (1944). A clinical syndrome associated with a rare anomaly of vena portal system. *Surgery, Gynecology & Obstetrics*.

[6] Baskan O., Erol C., Sahingoz Y. (2016). Portal biliopathy, magnetic resonance imaging and magnetic resonance cholangiopancreatography findings: a case series. *Gastroenterology Report*.

[7] Chawla Y., Dilawari J. B., Katariya S. (1994). Gallbladder varices in portal vein thrombosis. *American Journal of Roentgenology*.

[8] Kevans D., MacNicholas R., Norris S. (2009). Gallbladder wall variceal haemorrhage with associated rupture: a rare cause of mortality in the cirrhotic patient. *European Journal of Gastroenterology and Hepatology*.

[9] Vilallonga R., González O., Bergamini S., Fort J. M., Armengol M. (2012). Gallbladder variceal bleeding in a patient with alcoholic cirrhosis: a rare entity. *Revista Española de Enfermedades Digestivas*.

[10] Saad W. E. A., Lippert A., Saad N. E., Caldwell S. (2013). Ectopic varices: anatomical classification, hemodynamic classification, and hemodynamic-based management. *Techniques in Vascular and Interventional Radiology*.

[11] Chawla Y. K., Bodh V. (2015). Portal vein thrombosis. *Journal of Clinical and Experimental Hepatology*.

[12] Thrainsdottir H., Petursdottir V., Blöndal S., Björnsson E. S. (2014). Pancreatic mass leading to left-sided portal hypertension, causing bleeding from isolated gastric varices. *Case Reports in Gastrointestinal Medicine*.

[13] Garcia M. C., Ahlenstiel G., Mahajan H., Van Der Poorten D. (2015). Small bowel varices secondary to chronic superior mesenteric vein thrombosis in a patient with heterozygous Factor v Leiden mutation: a case report. *Journal of Medical Case Reports*.

[14] Haddad J. D., Lacey B. W. (2014). Isolated non-hemorrhagic cecal varices. *Gastroenterology Report*.

[15] Podboy A., Kamath P., Tabibian J. (2016). Isolated duodenal varices without cirrhosis. *The American Journal of Gastroenterology*.

[16] Arthur F. (1997). The cystic vein: the significance of a forgotten anatomic landmark. *JSLS: Journal of the Society of Laparoendoscopic Surgeons*.

[17] Walser E. M., Runyan B. R., Heckman M. G. (2011). Extrahepatic portal biliopathy: proposed etiology on the basis of anatomic and clinical features. *Radiology*.

[18] Petren T. (1932). Die extrahepatischen gallenwegsvenen and ihre: pathologischanatomische bedeutum. *Verhandlungen der Anatomischen Gesellschaft*.

[19] Khuroo M. S., Yattoo G. N., Zargar S. A. (1993). Biliary abnormalities associated with extrahepatic portal venous obstruction. *Hepatology*.

[20] Kim S. Y., Cho J. H., Kim E. J., Choi S. J., Kim Y. S. (2016). Successful hemostasis using a covered self-expandable metallic stent for spurting hemobilia in patients with advanced pancreatic cancer-induced portal biliopathy. *Gastrointestinal Endoscopy*.

[21] Dhiman R. K., Behera A., Chawla Y. K., Dilawari J. B., Suri S. (2007). Portal hypertensive biliopathy. *Gut*.

[22] Dilawari J. B., Chawla Y. K. (1992). Pseudosclerosing cholangitis in extrahepatic portal venous obstruction. *Gut*.

[23] Hellerich U., Pollak S. (1991). Spontaneous gallbladder rupture caused by variceal hemorrhage: an unusual complication of portal vein thrombosis. *Beiträge zur Gerichtlichen Medizin*.

[24] Pravisani R., Bugiantella W., Lorenzin D., Bresadola V., Leo C. A. Fatal hemoperitoneum due to bleeding from gallbladder varices in an end-stage cirrhotic patient A case report and review of the literature. *Annali Italiani di Chirurgia*.

[25] Chu E. C., Chick W., Hillebrand D. J., Hu K.-Q. (2002). Fatal spontaneous gallbladder variceal bleeding in a patient with alcoholic cirrhosis. *Digestive Diseases and Sciences*.

[26] Getzlaff S., Benz C. A., Schilling D., Riemann J. F. (2001). Enteroscopic cyanoacrylate sclerotherapy of jejunal and gallbladder varices in a patient with portal hypertension. *Endoscopy*.

[27] Mishin I. (2005). Gallbladder varices—a case report. *Romanian Journal of Gastroenterology*.

[28] Ralls P. W., Mayekawa D. S., Lee K. P., Colletti P. M., Johnson M. B., Halls J. M. (1988). Gallbladder wall varices: diagnosis with color flow Doppler sonography. *Journal of Clinical Ultrasound*.

[29] Palazzo L., Hochain P., Helmer C. (2000). Biliary varices on endoscopic ultrasonography: clinical presentation and outcome. *Endoscopy*.

[30] West M. S., Garra B. S., Horii S. C. (1991). Gallbladder varices: imaging findings in patients with portal hypertension. *Radiology*.

[31] Besa C., Cruz J. P., Huete A., Cruz F. (2012). Portal biliopathy: a multitechnique imaging approach. *Abdominal Imaging*.

[32] Özkavukcu E., Erden A., Erden I. (2009). Imaging features of portal biliopathy: frequency of involvement patterns with emphasis on MRCP. *European Journal of Radiology*.

[33] Chattopadhyay S., Nundy S. (2012). Portal biliopathy. *World Journal of Gastroenterology*.

